# Chk1 suppression leads to a reduction in the enhanced radiation-induced invasive capability on breast cancer cells

**DOI:** 10.1093/jrr/rrab049

**Published:** 2021-06-14

**Authors:** Takanori Adachi, Wantong Zhao, Kazumasa Minami, Yuhki Yokoyama, Daisuke Okuzaki, Rika Kondo, Yutaka Takahashi, Keisuke Tamari, Yuji Seo, Fumiaki Isohashi, Hirofumi Yamamoto, Masahiko Koizumi, Kazuhiko Ogawa

**Affiliations:** Department of Medical Physics and Engineering, Osaka University Graduate School of Medicine, 1-7 Yamadaoka, Suita, Osaka 565-0871, Japan; Department of Radiation Oncology, Osaka University Graduate School of Medicine, 2-2 Yamadaoka, Suita, Osaka 565-0871, Japan; Department of Radiation Oncology, Osaka University Graduate School of Medicine, 2-2 Yamadaoka, Suita, Osaka 565-0871, Japan; Department of Molecular Pathology, Osaka University Graduate School of Medicine, 1-7 Yamadaoka, Suita, Osaka 565-0871, Japan; Genome Information Research Center, Research Institute for Microbial Diseases, Osaka University Graduate School of Medicine, 3 Yamadaoka, Suita, Osaka 565-0871, Japan; Department of Medical Physics and Engineering, Osaka University Graduate School of Medicine, 1-7 Yamadaoka, Suita, Osaka 565-0871, Japan; Department of Radiation Oncology, Osaka University Graduate School of Medicine, 2-2 Yamadaoka, Suita, Osaka 565-0871, Japan; Department of Radiation Oncology, Osaka University Graduate School of Medicine, 2-2 Yamadaoka, Suita, Osaka 565-0871, Japan; Department of Radiation Oncology, Osaka University Graduate School of Medicine, 2-2 Yamadaoka, Suita, Osaka 565-0871, Japan; Department of Radiation Oncology, Osaka University Graduate School of Medicine, 2-2 Yamadaoka, Suita, Osaka 565-0871, Japan; Department of Molecular Pathology, Osaka University Graduate School of Medicine, 1-7 Yamadaoka, Suita, Osaka 565-0871, Japan; Department of Medical Physics and Engineering, Osaka University Graduate School of Medicine, 1-7 Yamadaoka, Suita, Osaka 565-0871, Japan; Department of Radiation Oncology, Osaka University Graduate School of Medicine, 2-2 Yamadaoka, Suita, Osaka 565-0871, Japan

**Keywords:** γ-ray, invasion, Chk1 inhibitor, breast cancer

## Abstract

Radiation therapy is generally effective for treating breast cancers. However, approximately 30% of patients with breast cancer experience occasional post-treatment local and distant metastasis. Low-dose (0.5 Gy) irradiation is a risk factor that promotes the invasiveness of breast cancers. Although an inhibitor of checkpoint kinase 1 (Chk1) suppresses the growth and motility of breast cancer cell lines, no study has investigated the effects of the combined use of a Chk1 inhibitor and radiation on cancer metastasis. Here, we addressed this question by treating the human breast cancer cell line MDA-MB-231 (*in vitro*) and mouse mammary tumor cell line 4 T1 (*in vitro* and *in vivo*) with γ-irradiation and the Chk1 inhibitor PD407824. Low-dose γ-irradiation promoted invasiveness, which was suppressed by PD407824. Comprehensive gene expression analysis revealed that low-dose γ-irradiation upregulated the mRNA and protein levels of S100A4, the both of which were downregulated by PD407824. We conclude that PD407824 suppresses the expression of S100A4. As the result, γ-irradiation-induced cell invasiveness were inhibited.

## INTRODUCTION

Radiation therapy, which confers significant benefits upon cancer treatments such as surgery and chemotherapy, has dramatically improved through the development of new technologies. For example, intensity-modulated radiation therapy delivers precise radiation doses to malignant tumors, thus reducing the damage to normal tissues adjacent to tumors [1]. Nevertheless, local recurrence and distant metastasis occasionally occur after treatment. In particular, the efficacy of radiation therapy is limited for treating metastasized tumors. For example, micrometastases may be present when radiation therapy commences or during treatment [2]. Unfortunately, few effective biological markers are available to guide radiation therapy and predict its efficacy. Further research efforts are therefore required, particularly those that target cancer metastasis.

Breast cancer frequently metastasizes to other organs, which accounts for the lower five-year relative survival rate (approximately 30%) of women with stage-IV of the disease compared with other stages [3]. Whole-breast radiation after breast-conserving surgery and chest-wall irradiation after mastectomy reduce the recurrence risk by approximately 11%, compared with no irradiation, and improve survival rates [4]. However, previous studies indicated that low-dose irradiation is one of the risk factors to promote the migration and invasiveness of breast cancer cells [5, 6].

Our previous study found that the checkpoint kinase 1 (Chk1) inhibitor PD407824 suppresses the growth and motility of triple-negative breast cancer cell lines as well as in an allograft model [7]. Irradiated cells undergo cell cycle arrest to repair the resultant DNA damage via Chk1-mediated signaling [8]. The Chk1 inhibitor AZD7762 blocks post-irradiation DNA repair, suggesting that the effects of radiation and this inhibitor are synergistic [9]. To the best of our knowledge, however, no study has attempted to show how the combined use of a Chk1 inhibitor and radiation affects cancer metastasis.

Here, we evaluated the effect of a Chk1 inhibitor and γ-irradiation on factors that influence breast cancer cell metastasis. We also investigated the mechanism underlying the effects.

## MATERIALS AND METHODS

### Cell culture

The human breast cancer cell line MDA-MB-231 and mouse mammary tumor cell line (4 T1) were cultured in Dulbecco’s Modified Eagle Medium (Thermo Fisher Scientific, Waltham, MA, USA) containing 10% fetal bovine serum (FBS) (Thermo Fisher Scientific) and 1% Penicillin–Streptomycin-Glutamine Mixed Solution (Nacalai Tesque, Kyoto, Japan) and maintained at 37°C in an atmosphere containing 5% CO_2_.

The schedule of experiments are summarized in Supplementary Fig. 1.

### Drug treatment

We selected PD407824 as the Chk1 inhibitor, referring our previous work (7). PD407824 (C_20_H_12_N_2_O_3_) (Tocris Bioscience, Bristol, UK) [10] was dissolved in dimethyl sulfoxide (DMSO) (Wako, Osaka, Japan) and stored at 4°C. After 2 h, the medium containing 10% FBS was replaced with the same medium containing PD407824.

### Transfection

A Silencer Select siRNA targeting Chk1 (siChk1) (Thermo Fisher Scientific) and a Silencer Select negative control No. 2 siRNA (siNC) (Thermo Fisher Scientific) were used to transfect cells in the presence of Lipofectamine RNAiMAX and Opti-MEM (Thermo Fisher Scientific) in the absence of antibiotics following manufacture protocol. Immediately after irradiation, antibiotic-free medium was replaced with the same medium but containing 10% FBS.

### Irradiation

Cells were irradiated with the γ-ray emitter cesium-137 (^137^Cs) using a Gammacell 40 Exactor (MDS Nordion, Ottawa, Canada). The dose rate was approximately 0.81 Gy/min.

### MTT assay

The MTT Cell Proliferation Assay Kit (Trevigen, Gaithersburg, MD, USA) were used to measure cell proliferation. Immediately after irradiation, 4000 cells with Dulbecco’s Modified Eagle Medium containing 10% FBS and 1% Penicillin–Streptomycin-Glutamine Mixed Solution were added to the wells of 96-well cell culture plates. After irradiation (2, 24, 48, and 72 h), an MTT reagent was added to each well (10 μL/well), and the cells were incubated for 3 h at 37°C after covering the plates with aluminum foil. After incubation, the kit’s Detergent Reagent was added to each well (100 μL/well), and the cells were incubated for a further 3 h at room temperature. Absorbance of 562 nm was measured using a Multiskan GO Microplate Spectrophotometer (Thermo Fisher Scientific).

### Colony formation assay

Immediately after irradiation, cells were trypsinized, added to 60-mm culture dishes, cultured for 14 days, fixed with 10% formalin, and stained with 0.04% crystal violet. At least 50 cells in each colony were counted as surviving cells. Plating efficiency was calculated by the formula, (Number of colonies counted)/(Number of cells seeded) × 100. And the surviving fraction (SF) was calculated relative to unirradiated cells.

### Wound healing assay

Cells were grown to confluence in 60-mm culture dishes, and the cell monolayer was scratched using a plastic pipette tip. Areas newly occupied with cells in the scratch zone were measured 24 h after irradiation. The plates were observed using a fluorescence microscope (×200 magnification), and the scratch zone were analyzed using ImageJ [11].

### Matrigel invasion assay

The invasiveness of cells was measured using Chemotaxicell filters (Kurabo Industries, Osaka, Japan) coated with Matrigel (Corning, NY, USA). Twenty-four hours after irradiation, cells were suspended in serum-free medium containing 0.1% bovine serum albumin (BSA) (Wako). Cells were added to the upper well (2 × 10^5^ cells/well), and medium containing 10% FBS (chemoattractant) was added to the lower well. Cells were incubated for 24 h, fixed with 10%-buffered formalin, and then stained with hematoxylin and eosin (H&E). The number of cells on the lower side of the membrane was counted using a fluorescence microscope (×200 magnification).

### Real-time qPCR

Total RNA for qPCR analysis was extracted using an RNeasy Plus Mini Kit (Qiagen, Germantown, MD, USA) and reverse-transcribed using a High-Capacity cDNA Reverse Transcription kit (Applied Biosystems, Carlsbad, CA, USA). Real-time qPCR was performed using a Power SYBR Green PCR Master Mix Kits (Applied Biosystems) with the PCR primers as follows: forward and reverse primers, respectively, were 5′-GGGCAAAGAGGGTGACAAGTTC-3′ and 5′-CTGGGCTGCTTATCTGGAAG-3′ for S100A4; 5′-CTCAGATCCGTGGTGAGATCT-3′ and 5′-CTTTGGTTCTCCAGCTTCAGG-3′ for MMP-2; and 5′-GGAAATCGTGCGTGACA-3′ and 5′-TCAGGAGGAGCAATGATC-3′ for β-actin, respectively. After confirming the amplification efficiency and linearity of amplification, analysis was performed. Values were calculated using the 2^−ΔΔ*CT*^ method and normalized to those of β-actin.

### Western blot analysis

Twenty-four hours after irradiation, cell lysates were prepared using RIPA buffer (Santa Cruz Biotechnology, Dallas, TX, USA). Proteins were fractionated using 10–15% SDS polyacrylamide gradient gels, and the proteins were electrophoretically transferred to PVDF membranes (Millipore, Burlington, MA, USA). Primary antibodies were against S100A4 (Sheep IgG, Cat. # AF4138, R&D Systems, Minneapolis, MN, USA), p-Chk1 (Ser345) (Rabbit IgG, Cat. # 2348, Cell Signaling, Danvers, MA, USA), Chk-1 (Mouse IgG, Cat. # 2360, Cell Signaling) (dilute to 1:1000), and β-actin (Mouse IgG, Cat. # ab8226, Abcam, Cambridge, UK) (dilute to 1:10000). Secondary antibodies were used Goat pAb to Ms IgG (HRP) (Cat. # ab97023, Abcam) and Goat pAb to Rb IgG (HRP) (Cat. # ab97051, Abcam) at a 1:2000 dilution. PD407824 inhibited the phosphorylation and activity of Chk1-Ser345 [12], and siChk1 downregulated the expression of Chk1 and p-Chk1 [13]. Membranes were washed in PBST, and luminescence detected using Pierce ECL Western Blotting Substrate (Thermo Fisher Scientific) was measured using an ImageQuant LAS 4000 (GE Healthcare, Little Chalfont, UK).

### Matrix metalloproteinase-2 (MMP-2) activity assay

Serum-free medium containing DMSO or PD407824 was used to replace the culture medium (2 mL). Cells were irradiated after 4 h incubation and incubated further for 24 h at 37°C. An MMP2 activity assay kit (AnaSpec, CA, USA) was used according to the source’s protocol. MMP-2 activity was spectrophotometrically measured at 520 nm using a Varioskan LUX (Thermo Fisher Scientific).

### Mouse model

The Animal Experiment Committee of our institution approved the protocol for using mice. Three female BALB/c mice (six-weeks-old; Nihon-Clea, Tokyo, Japan) were housed per cage with free access to food and water. Four T1 cells, described above, were subjected to 0.5 Gy γ-irradiation (IR cells) or incubated using the same conditions without irradiation (non-IR cells). Dead cells (nonadherent) were identified after 72 h and removed from the culture dishes. On day 1, the tail veins of mice were inoculated with IR or non-IR cells (1.0 × 10^5^ cells in 50 μL PBS). PD407824 or vehicle was injected i.p. each day (2 mg/kg body weight). The mice were killed on day 21 and their lungs were harvested. We counted the number of metastatic nodules on the surface of the lung. H&E staining was conducted to evaluate the micrometastatic area in the lung. The metastatic area were calculated using ImageJ. These results were expressed as a relative value base on the non-irradiated group. The schedule of the experiment is summarized in Supplementary Fig. 2.

### Statistical analysis

The results were expressed as mean values with standard deviations. These were followed by a normal distribution and equality of variance. Therefore, the statistical significance was evaluated using the Student *t* test, and *P* < 0.05 indicates a significant difference.

## RESULTS

### PD407824 suppresses motility and radiation-induced invasiveness of MDA-MB-231 cell line

**Fig. 1.**
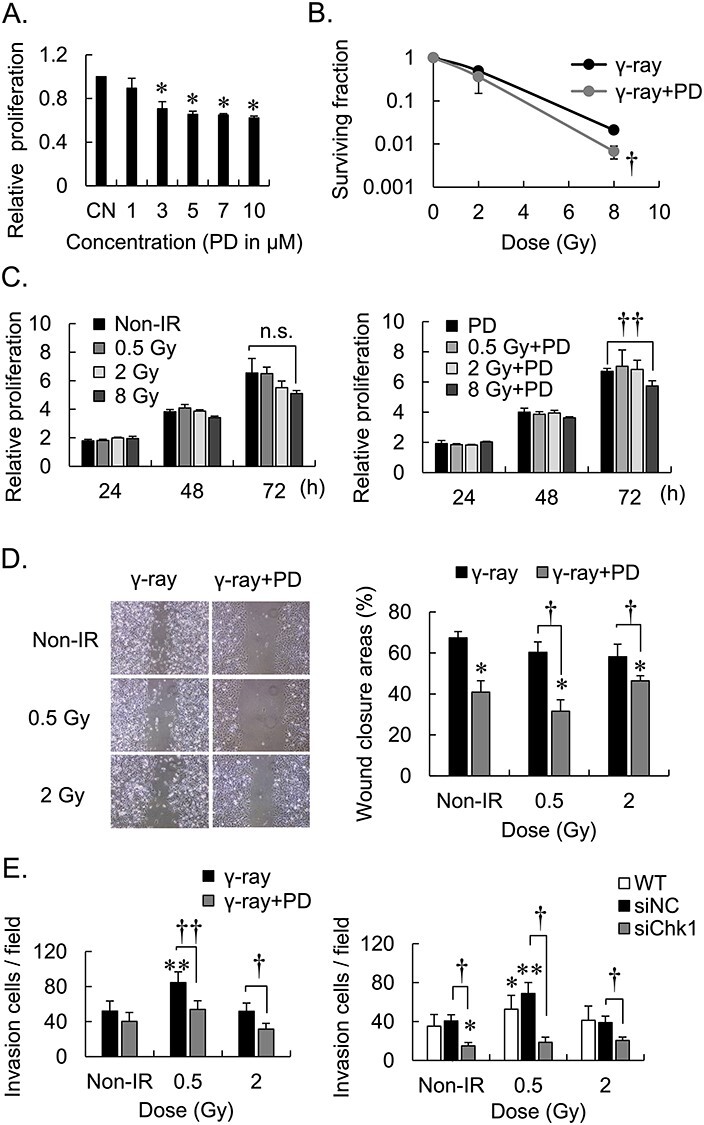
Combined effects of the Chk1 inhibitor PD407824 and γ-irradiation of the survival, proliferation, motility, and invasiveness of MDA-MB-231 cell line. CN = control, WT = wild type, Non-IR = no irradiation, PD = PD407824; ^*^P < 0.05, ^*^^*^P < 0.01 vs CN, WT or Non-IR, respectively; ^†^*P* < 0.05 and ^††^*P* < 0.01, respectively; n.s. not a significant difference; error bar means standard deviation. All experiments conducted three times independently: (i) MTT assays of cells treated with PD407824, (ii) Effects of PD407824 on Colony formation by γ-irradiated cells, (iii) MTT assays of the proliferation of cells treated with γ-irradiation plus PD407824, (iv) Effects of PD407824 on cell motility in wound healing, (v) Matrigel invasion assay of cells treated with PD407824.

The MTT assay was employed to determine the cytotoxicity of PD407824 to MDA-MB-231 cell line (Fig. 1a). There were significant differences in cell proliferation in the presence of <3 μM PD407824 vs the vehicle (1.26 fold, *P* = 0.027). We therefore used 1 μM PD407824 to provide a safe margin to avoid cytotoxicity.

The Colony formation assays were employed to evaluate the radiosensitive effects of PD407824 on irradiated MDA-MB-231 cells (Fig. 1b). PD407824 significantly enhanced radiation sensitivity when cells were irradiated with 8 Gy (3.1 fold, *P* = 0.031). We assessed the combination effect of PD407824 and radiation on cell proliferation by MTT assay (Fig. 1c). The combination of PD407824 and irradiation (24 h and 48 h) did not significantly affect cell proliferation compared with controls. However, 72 h after irradiation with 8 Gy, cell proliferation was significantly inhibited after treatment with PD407824 (1.1 fold, *P* = 0.0068).

We next used a wound healing assay to evaluate the effects of irradiation on cell motility for as long as 24 h (Fig. 1d). The motility of cells irradiated with 0.5 Gy was not significantly different to that of unirradiated cells. PD407824 significantly suppressed motility (1.65 fold, *P* = 0.037), regardless of whether cells were irradiated.

To evaluate the invasiveness of MDA-MB-231 cells, we performed a Matrigel invasion assay 24 h after irradiation (Fig. 1e). In contrast to the results of the wound healing assay, irradiation with 0.5 Gy increased the invasiveness of irradiated cells (1.56 fold, *P* = 0.0029) but not that of unirradiated cells. Further, MDA-MB-231 cells transfected with siChk1 and treated with PD407824 were less invasive after irradiation compared with siNC transfected cells.

### PD407824 and low-dose irradiation is associated the expression of S100A4 cell invasion associated gene

**Fig. 2.**
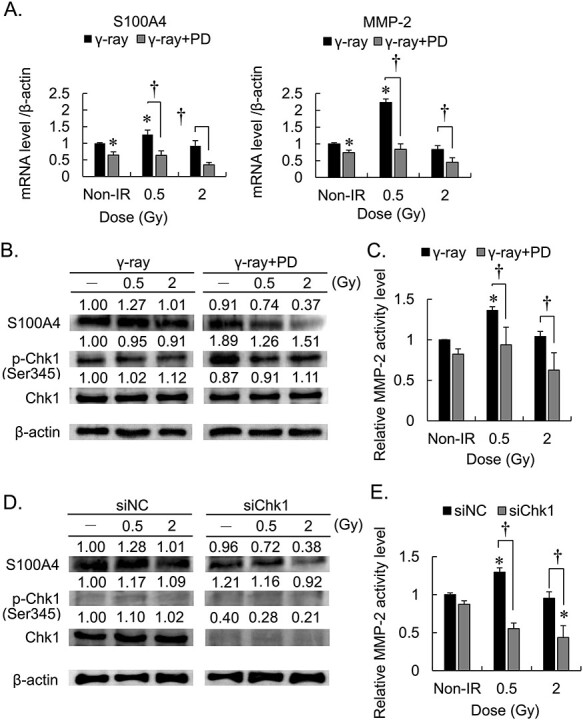
Combined effects of PD407824 and γ-irradiation on the expression and activities of S100A4 and MMP-2 on MDA-MB-231 cells. Non-IR = no irradiation, PD = PD407824. ^*^P < 0.05 vs Non-IR ^†^*P* < 0.01; error bar means standard deviation. All experiments conducted three times independently. (A) Effects of PD407824 on S100A4 and MMP-2 mRNA levels. (B) Effects of PD407824 combined with γ-irradiation on S100A4 and phosphorylation of Chk1. (C) Effect of PD407824 on MMP-2 activity. (D) Effects of a Chk1-specific siRNA (siChk1) on S100A4 and p-Chk1 expression. (E) Effect of siChk1 on MMP-2 activity.

We focused on the S100 family of calcium-binding proteins and matrix metalloproteinases (MMPs) that are associated with the invasiveness of breast cancer cells [7, 14]. We performed qRT-PCR assays to measure the levels of the mRNAs encoding S100A4 and MMP-2 (Fig. 2A). The levels of S100A4 and MMP-2 mRNAs of 0.5 Gy irradiated cells were significantly higher compared with those of unirradiated cells (1.35 fold, *P* = 0.031 and 2.3 fold, *P* = 0.028, respectively.). PD407824 inhibited the expression of both mRNAs independent of whether the cells were irradiated.

Western blot analysis of S100A4 expression and MMP-2 activity assays were conducted 24 h after irradiation (Fig. 2b and c). Compared with the controls, the levels of S100A4 expression and MMP-2 activity were elevated in cells exposed to 0.5 Gy (1.36 fold, *P* = 0.029). However, these increases were reduced when the cells were exposed to 0.5 Gy or 2 Gy after treatment with PD407824. Chk1 knockdown cells also were suppressed the expression of S100A4 protein and MMP-2 activity by irradiation after treatment with PD407824 (Fig. 2d and e).

### PD407824 suppresses lung metastasis caused by 4 T1 cells exposed to low-dose γ-irradiation

**Fig. 3.**
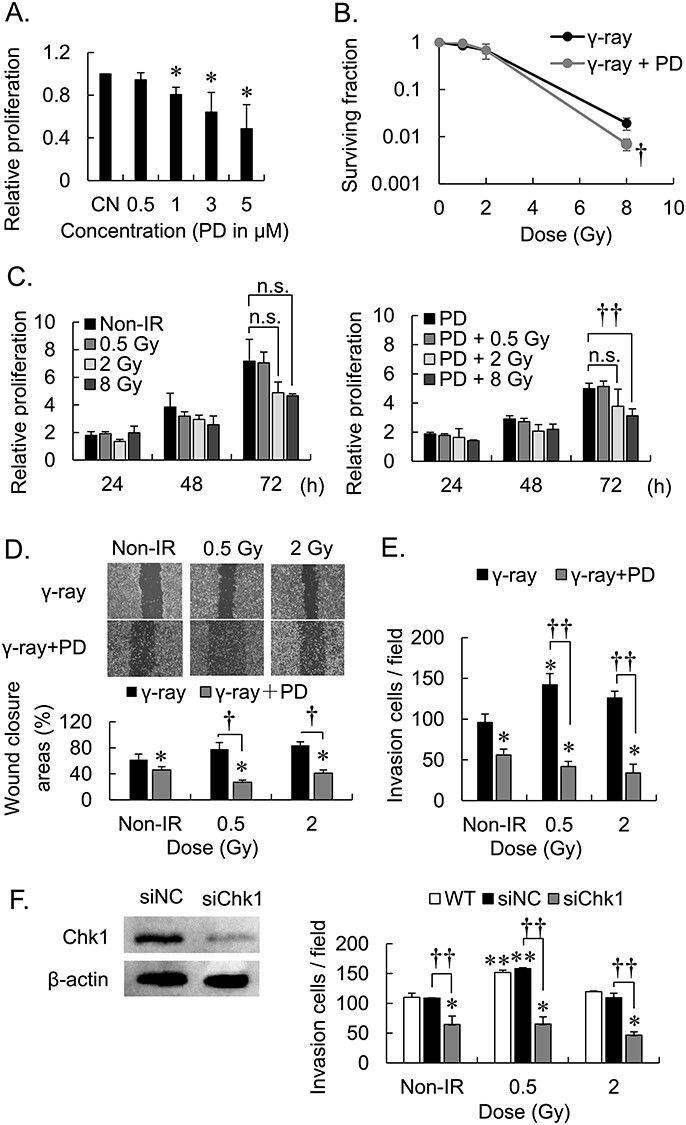
Combined effects of the Chk1 inhibitor PD407824 and γ-irradiation of the survival, proliferation, motility, and invasiveness of 4 T1 cell line. CN = control, WT = wild type, Non-IR = no irradiation, PD = PD407824; ^*^P < 0.05, ^*^^*^P < 0.01 vs CN, WT or Non-IR, respectively; ^†^*P* < 0.05 and ^††^*P* < 0.01, respectively; n.s. not a significant difference; error bar means standard deviation. All experiments conducted three times independently. (A) MTT assays of cells treated with PD407824. (B) Effects of PD407824 on Colony formation by γ-irradiated cells. (C) MTT assays of the proliferation of cells treated with γ-irradiation plus PD407824. (D) Effects of PD407824 on cell motility in wound healing. (E) Matrigel invasion assay of cells treated with PD407824. (F) Matrigel invasion assay of Chk1 knock down cells treated with PD407824.

Before conducting the experiment in mouse model, the cytotoxicity, the cell survival, proliferation, motility, and invasiveness of the 4 T1 cell line were evaluated. The proliferation of 4 T1 cells significantly decreased after treatment with 1 μM PD407824 (Fig. 3A). Therefore, we treated cells in the following experiments with 0.5 μM PD407824. The effects of PD407824 on the results of Colony formation, MTT, Wound healing and Matrigel invasion assays of 4 T1 cells and MDA-MB-231 cells were similar (Fig. 3b–f).

**Fig. 4.**
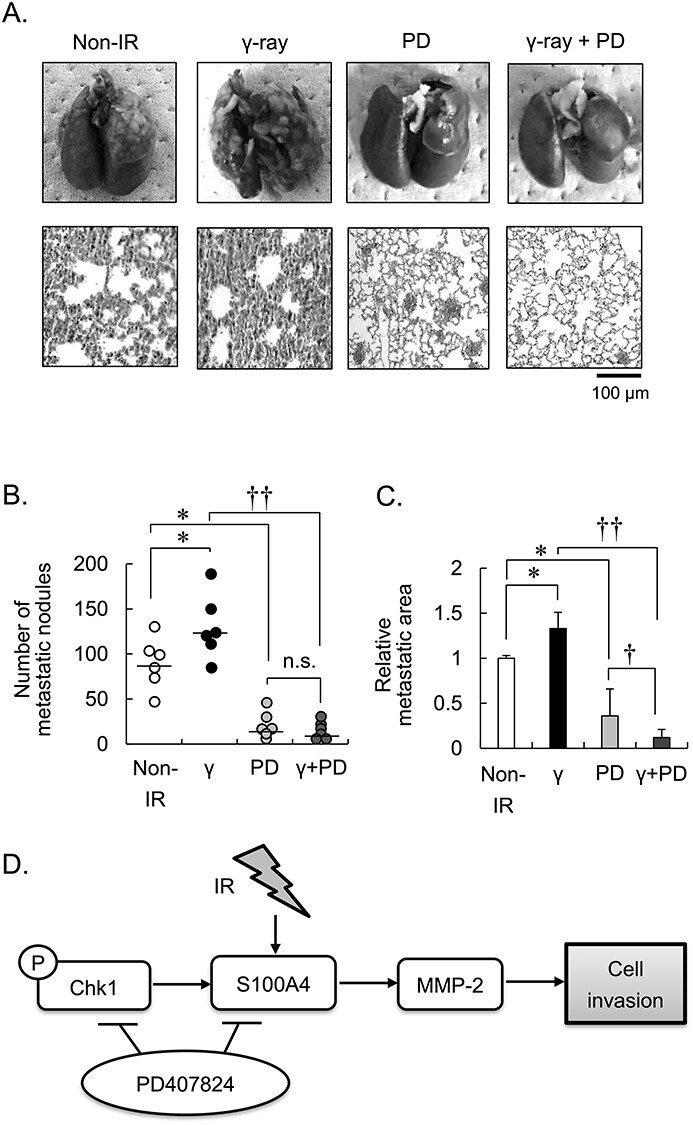
Mouse model to evaluate the effects of PD407824 and γ-irradiation on metastasis. Non-IR = no irradiation, γ = γ-radiation and PD = PD407824; ^*^P < 0.05 vs CN or Non-IR; n.s. not a significant difference; ^†^*P* < 0.01; error bar means standard deviation. Six mice belongs for each groups. Effects of PD407824 on the metastatic progression of transplanted 4 T1 cells. (A) The images of lung surface and HE staining of lung section in each groups. (B) The number of metastatic nodules on lung surface. (C) The relative metastatic area of lung section.

The mice belonged to one of four groups: Non-IR, γ-ray, PD, or γ-ray + PD. We showed the picture of lung of four groups (Fig. 4a). We counted the number of metastatic nodules on the surface of the lung (Fig. 4b). There were significantly more metastatic nodules in the lungs of mice inoculated with cells exposed to γ-irradiation (γ-ray group) compared with Non-IR mice (1.43 fold, *P* = 0.041). In contrast, the number of metastatic nodules in the lungs of mice inoculated with IR cells or non-IR cells, which were then injected with PD407824 (γ-ray + PD or PD group), were significantly reduced (0.18 fold, *P* = 0.018 and 0.21 fold, *P* = 0.020, respectively.). H&E staining showed that the metastatic area of lungs on PD-treated mice were reduced with or without γ-irradiation groups. Notably, the γ-ray + PD group mice had the lowest metastatic area of their lungs (0.33 fold compared with PD group) (Fig. 4c).

## DISCUSSION

The study showed that low-dose irradiation (0.5 Gy) promoted the cell invasiveness and metastasis of breast cancer cell lines, and that the former was suppressed by the Chk1 inhibitor PD407824 *in vitro* and *in vivo*. The results of the siRNA experiment targeting Chk1 indicate that Chk1, not the pharmacological effect of PD407824, was significantly associated with cell invasiveness.

*In vitro* experiments, MDA-MB-231 cells and 4 T1 cells were used to evaluate the differential effects of Chk1 inhibitor on the two cell lines (Figs 1 and 3). To determine whether cell viability affected the results of the Matrigel invasion assays, we focused on cell proliferation and survival. Our results repealed that within 72 h after irradiation of cells with doses of 0.5 Gy and 2 Gy, there were no significant differences, indicating that the combination of PD407824 and 2 Gy-irradiation did not have a significant influence on cell phenotype. However, the combination of PD407824 and an 8-Gy dose of irradiation suppressed cell proliferation after 72 h. Moreover, the results of the Colony formation assay were consistent with previous study regarding the effect of Chk1 inhibitor on radiosensitivity [9]. Accordingly, we suggest that the combined use of PD407824 and high-dose irradiation contributes to radiosensitivity and that PD407824 combined with low-dose irradiation significantly affects cell invasion.

We focused on S100A4, which belongs to the S100 family of calcium-binding proteins that are required for cell survival, angiogenesis, and metastasis [15]. For example, upregulating the expression of S100A4 promotes cancer metastasis and correlates with poor prognosis of multiple cancers [16, 17]. In addition, the increase in MMP-2 activity enhances cell invasion via the regulation of S100A4 [18, 19]. Here, our results showed that the combined use of a Chk1 inhibitor and low-dose irradiation decreased the activities of MMP-2 and expression of S100A4 proteins (Fig. 2b–e). S100A4 is detected in the nucleus, cytoplasm, and extracellular space, and S100A4 in the nucleus promotes cell invasion [20, 21]. Thus the Chk1 inhibitor PD407824 and low-dose irradiation may regulate cell invasiveness via gene and protein expression of S100A4.

Numerous studies have focused on members of the S100 protein family and their potential to serve as targets of therapy for inflammatory conditions, Alzheimer’s disease, and cancer metastasis [16, 17, 22]. In particular, S100A4 may serve as a target of therapies to prevent, control, or ablate cancer metastasis. Therefore, the expression of S100A4 may predict the response of metastatic tumor cells to irradiation and treatment with a Chk1 inhibitor.

There were some limitations in this study. First, we did not show the data on the effects of Chk1 inhibitor on other kinds of breast cancer and normal cells. When considering the clinical approach, this is an important point. Next, we have not shown the data using cells that have been transfected with S100A4. Hence, it is not clear that Chk1 inhibitor suppressed the invasiveness of breast cancer cells via just the expression of S100A4. And we focused our study on the conditions up to 2 Gy to investigate pure invasion and protein expression analysis without the effect of cell death. In clinic, it is valuable to evaluate the above analysis including cell death. Third, the molecular functions of cell cycle-related proteins including Chk1 are poorly understood. Further studies are required to identify other off-target effects of Chk1 inhibitors.

In summary, to the best of our knowledge, our study is the first to confirm that a Chk1 inhibitor combined with γ-irradiation suppresses the invasiveness of breast cancer cell lines, resulting from inhibiting the expression of S100A4. The efficacy of combination therapy strongly correlates with both its effect on cancer cell metastasis, as well as on radiosensitivity-associated cell cycle checkpoint-mediated effects. Therefore, Chk1, when combined with radiation therapy, may serve as a therapeutic target for killing metastatic cancer cells.

## Supplementary Material

Supplementary_Figure_1_rrab049

Supplementary_Figure_2_rrab049

## PRESENTATION AT A CONFERENCE

The 24^th^ Seminar for Sensitization of Cancer Treatment and the 7^th^ International Symposium of Training Plan for Oncology Professionals.
